# Recipes

**Published:** 1861-07

**Authors:** 


### Recipes.

We have no decided partiality for special formulas. The routinish prescription of particular combinations of drugs, for the cure of a given disease, without due regard to the properties of each ingredient, and the special indications in the case, leads too often to confirmed empiricism difficult to be overcome. This habit is decidedly pernicious in the young practitioner, while fixing in his mind those great principles which are to guide him through his professional career. And while the happy and judicious combination of medicinal agents, to effect certain changes in the system, should not be ignored, but are with propriety, to a certain extent, studied and preserved at the same time, caution should be observed, lest the abominable habit of associating the *name* of a remedy so intimately with the *name* of a disease that the rational application of medicines for the cure of disease, is lost sight of.

In the present issue will be found, in addition to some useful unofficinal simple preparations, several recipes for compounds, as the author properly intimates, for the convenience of those requiring the combined action of the articles thus associated.

